# Monoballism Associated with Newly Onset Ketotic Hyperglycemia

**DOI:** 10.1155/2012/202708

**Published:** 2012-12-26

**Authors:** Dilek Ersil Soysal, Barıs Gelen, Sezin Hızar, Mete Pekdiker, Ebru Tekesin, Yesim Beckmann, Volkan Karakus

**Affiliations:** ^1^Department of Internal Medicine, University of Katip Celebi and Ataturk Research and Training Hospital, Basın Sitesi, Izmir 35360, Turkey; ^2^Department of Neurology, University of Katip Celebi and Ataturk Research and Training Hospital, Basın Sitesi, Izmir 35360, Turkey

## Abstract

Movement disorders as the initial symptoms of diabetes mellitus are rare. Here, we describe one of these rare manifestations of primary diabetes: a case of newly diagnosed diabetes mellitus in an old-age female patient with transient monoballismus during an episode of ketotic hyperglycemia.

## 1. Introduction

Hemichorea-hemiballismus (HC-HB) constitute a neurological syndrome characterized by violent proximal involuntary movements on one side of the body, involving mainly the upper extremity [1]. Focal epilepsy, transient chorea or ballism provoked by an episode of nonketotic hyperglycemia (NKH) in adults with type 2 diabetes [1–8], and ketotic hyperglycemia in children with type I diabetes mellitus have been reported [9]. Nonketotic hyperglycemia occurs more often in women [1, 3, 6] and usually is associated with very high blood glucose [3]. In these cases, the seizures [7] as well as the choreiform movements have resolved within days to a few weeks after normalization of blood glucose and hence, reversible metabolic derangements within the basal ganglia have often been assumed [1–4, 8, 9]. Most of the cases have MRI changes in the putamen with high signal intensity on T1-weighted images and variable signal characteristics ranging from hyper-, to iso-, to hypointensity on T2-weighted images [1–3, 6, 8].

Movement disorders as the initial symptoms of diabetes mellitus are rare [5, 8]. Here, we describe one of these rare manifestations of primary diabetes: a case of newly diagnosed diabetes mellitus in an old-age female patient with transient monoballismus during an episode of ketotic hyperglycemia.

Ballism can be rapidly controlled by normalization of glycemia. Our patient had monoballism confined to her upper extremity. To our knowledge, this is the first report describing monoballism in a patient with ketotic hyperglycemia. She had a rapid symptomatic remission after correction of the hyperglycemia.

## 2. Case Presentation

An 84-year-old woman was brought to the emergency department with a complaint of acute onset, rapid, involuntary movements of the left upper limb and she was diagnosed with ketotic hyperglycemia with positive urine ketones. She had no significant past medical history except for essential hypertension. The patient was not exposed to newly onset medications, but was receiving angiotensin receptor blocker (valsartan 160 mg/d, orally) for hypertension. There was no family history of movement disorders, and she had no previous neurological and psychiatric symptoms either. On admission, the general physical examination was normal. Neurological examination showed an alert patient with acute onset involuntary, continuous, jerky movements of the left arm. The rest of the neurologic examination was unremarkable. The random blood glucose concentration was 309 mg/dL with (++) urine ketones and glycosuria. She had normal arterial blood gas analysis of pH, P_CO_2__ and HCO_3_^−^ concentrations on room air and temperature. Blood count and liver function tests were normal. Her urea was 25 mg/dL and creatinine 1.21 mg/dL. Her glycosylated hemoglobin A1C (HbA1C) was 14.9%. Serum sodium: 130 mEq/L, potassium: 3.9 mEq/L, serum calcium: 8.6 mg/dL, phosphorus: 2.3 mg/dL, magnesium: 1.7 mg/dL, uric acid: 6.1 mg/dL, serum ceruloplasmin: 30.6 mg/dL (N: 20–60), and copper:135 *μ*g/dL (N: 80–155). Estimated blood osmolality was 294 mosm/L (N: 285–295). Antinuclear antibody (ANA) profile, antineutrophilic cytoplasmic antibodies reactive to myeloperoxidase (p-ANCA), and proteinase-3 (c-ANCA), antiphospholipid antibodies (e.g., anticardiolipin antibodies), antismooth muscle antibody (ASMA), anti-liver-kidney-microsome antibody (LKM-1), antimitochondrial antibody (AMA), TSH receptor antibodies (TRab), antithyroid peroxidase (TPO), and antithyroglobulin (anti T) antibodies revealed negative. Chest radiograph and electrocardiograph were normal.

**Figure 1:**
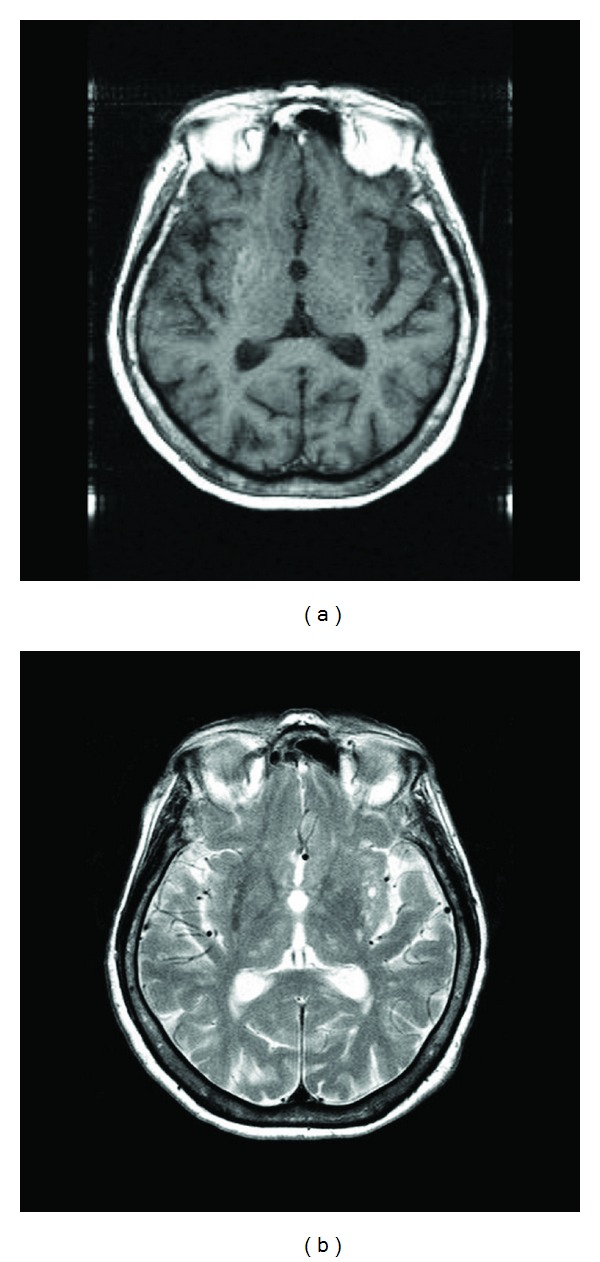
(a) MRI scan showing a hyperintense lesion on T1-weighted image. Arrow corresponds to the right striatum. (b) MRI scan showing a low signal intensity on T2-weighted image.

Brain magnetic resonance imaging (MRI) showed hyperintensity in the right striatum on T1-weighted (Figure 1(a)) and low signal intensity on T2-weighted images (Figure 1(b)).

Management for diabetic ketosis was started on intravenous (IV) insulin infusion with hourly blood sugar monitoring. After the patient's glycemic control was achieved and urine ketones were negative, treatment with rapid-acting analog insulin of 8 unit before meals and long-acting analog insulin of 12 unit at night subcutaneously, and quetiapine fumarate 25 mg/d orally were commenced on the second day of her admission.

After achievement of blood glucose levels between 79 and 141 mg/dL, the involuntary movements declined and completely disappeared within 6 days, and quetiapine fumarate was discontinued without any recurrence of the described movements. The patient was discharged on a combination of rapid-and-long acting analog insulin (10 + 10 + 10 + 16 U SC) and advised regular follow up for glycemic profiles.

## 3. Discussion

“Ballism” refers to violent, irregular flinging limb movements. It constitutes part of the spectrum of chorea and is a result of proximal muscle contraction. There are several types of ballism, depending on the distribution of movements. The most common form is “hemiballism,” which involves flinging movements of the upper and lower extremities on one side of the body. Ballism confined to one extremity is referred to as “monoballism.” [10]. Chorea or ballismus can be caused by a variety of disorders affecting the basal ganglia such as metabolic diseases (it is possible for a patient with chorea-ballismus to have hyperglycemia at the initial presentation) [1, 6, 8], hypoxic-ischemic events, vascular disorders, structural abnormalities, trauma, drugs and toxins, infections, and inflammatory immunological diseases (rheumatic fever-Sydenham's chorea, systemic lupus erythematosus) [1, 4, 5, 8, 9].

Our patient suffered from monoballismus that was associated with an episode of ketotic hyperglycemia of an undiagnosed diabetes. She recovered quickly after correction of the hyperglycemia and treatment with quetiapine fumarate. Lack of recurrence of the involuntary movements after cessation of the drug suggested that this disorder was related to the metabolic change and that acute chorea-ballismus caused by hyperglycemia was a treatable disorder with a good prognosis [1, 4, 5, 8, 9].

The pathogenesis of ballismus associated with hyperglycemia is poorly understood. In ketoacidosis, ketones are used (an abundant source of acetoacetate from which *γ*-aminobutyric acid might be resynthesised) as an energy source and *γ*-aminobutyric acid (GABA) can be produced, but in nonketotic patients GABA will be rapidly depleted [5]. Depletion of GABA may cause a decrease in GABAergic activity, thereby decreasing the inhibition of the thalamus by the medial part of the globus pallidus [1]. As a result, HC-HB or partial seizures rarely occur with diabetic ketoacidosis. In nonketotic hyperglycemia, the brain metabolizes GABA into succinic acid via the succinic acid semialdehyde pathway and thus depletes GABA rapidly [4, 5]. During hyperglycemia, the activity of tricarboxylic acid cycle (Krebs cycle) and glucose utilization are depressed in the brain, so the cerebral metabolism shifts to alternative pathways [4]. In nonketotic hyperglycemia, the shift to anaerobic metabolism causes brain to utilize amino butyric acid which is synthesized from acetoacetate. Unlike in ketoacidosis, acetoacetate is rapidly depleted in nonketotic hyperglycemia causing cellular dysfunction [4, 5, 9].

Most reported cases of hyperglycemia-associated chorea and ballism are elderly, predominantly female diabetic patients with nonketotic hyperglycemia, and of East Asian origin [6] like in our case (except that the patient was ketotic hyperglycemia which was a rare condition), and the presentation is usually acute with the choreic-ballistic movements emerging as hyperglycemia develops. The female predisposition may be related to postmenopausal alterations of GABA or dopamine receptors [1]. Our patient had monoballism confined to her upper extremity. To our knowledge, this is the first report describing monoballism in a patient with ketotic hyperglycemia.

The patient's brain MRI showed a unilateral hyperintense lesion on T1-weighted images, and low signal intensity on T2-weighted images in the right striatum. Unilateral involvement in chorea induced by nonketotic hyperglycemia is more common in the literature than generalized chorea, which is the opposite of what we should expect from a central nervous system disorder provoked by a metabolic abnormality [2, 6]. Also, putamen is the most consistent focus in hyperglycemia-related chorea [2, 8]. A question is raised whether this clinical syndrome is due to selective metabolic vulnerability of the striatum to hyperglycemia and the attendant fluid and electrolyte abnormalities in predisposed individuals [2]. A recent case report on HC-HB assumed that diabetic ketoacidosis could cause this syndrome as a result of osmotic shifts generated by rapid changes in serum glucose levels and the putaminal lesion that was seen on T1-weighted imaging probably resulted from a diverging response to the same hyperglycemia [11].

Chorea-ballism can be rapidly controlled by normalization of glycemia [5, 6]. Resolution of the lesions seen in imaging studies was found to be slower than the clinical course [6, 8, 11]. Our patient had a rapid symptomatic remission after correction of hyperglycemia. In some cases, involuntary movements can persist for a longer period despite correction of hyperglycemia [2, 6], and these patients may respond to conventional neuroleptics, particularly haloperidol, perphenazine, and chlorpromazine; risperidone and anticonvulsants may also be useful [6]. 

## 4. Conclusion

Even though numerous cases of hyperglycemia-provoked chorea-ballism have been described since its first report in 1960, it is uncommon in clinical practice [6]. 

Ballism is rarely caused by a dysfunction of glucose metabolism. Checking blood glucose in patients with this type of hyperkinesia, particularly in older women, is advised as the condition may rapidly resolve with hydration and resolution of the hyperglycemia. It is also advisable to keep in mind that this movement disorder may be the initial presenting symptom of diabetes mellitus [5].
